# Supplementary material for: Location-specific co-benefits of carbon emissions reduction from coal-fired power plants in China
Source: Nat Commun. 2021 Nov 29;12:6948. doi: 10.1038/s41467-021-27252-1 (PMC8629986; doi:10.1038/s41467-021-27252-1)
Supplement: Supplementary file 1 — Supplementary information [file 41467_2021_27252_MOESM1_ESM.pdf]

Supplementary information

Location-specific co-benefits of carbon emissions reduction from coal-fired power plants in China

Pu Wang*, Cheng-Kuan Lin, Yi Wang*, Dachuan Liu, Dunjiang Song, Tong Wu

*Corresponding authors:
Pu Wang, Institutes of Science and Development, Chinese Academy of Sciences. No.15 Zhongguancun Beiyitiao Alley, Haidian District, Beijing, 100190, China. Email: wangpu@casisd.cn.
Yi Wang, Institutes of Science and Development, Chinese Academy of Sciences. No.15 Zhongguancun Beiyitiao Alley, Haidian District, Beijing, 100190, China. Email: wangyi@casisd.cn.

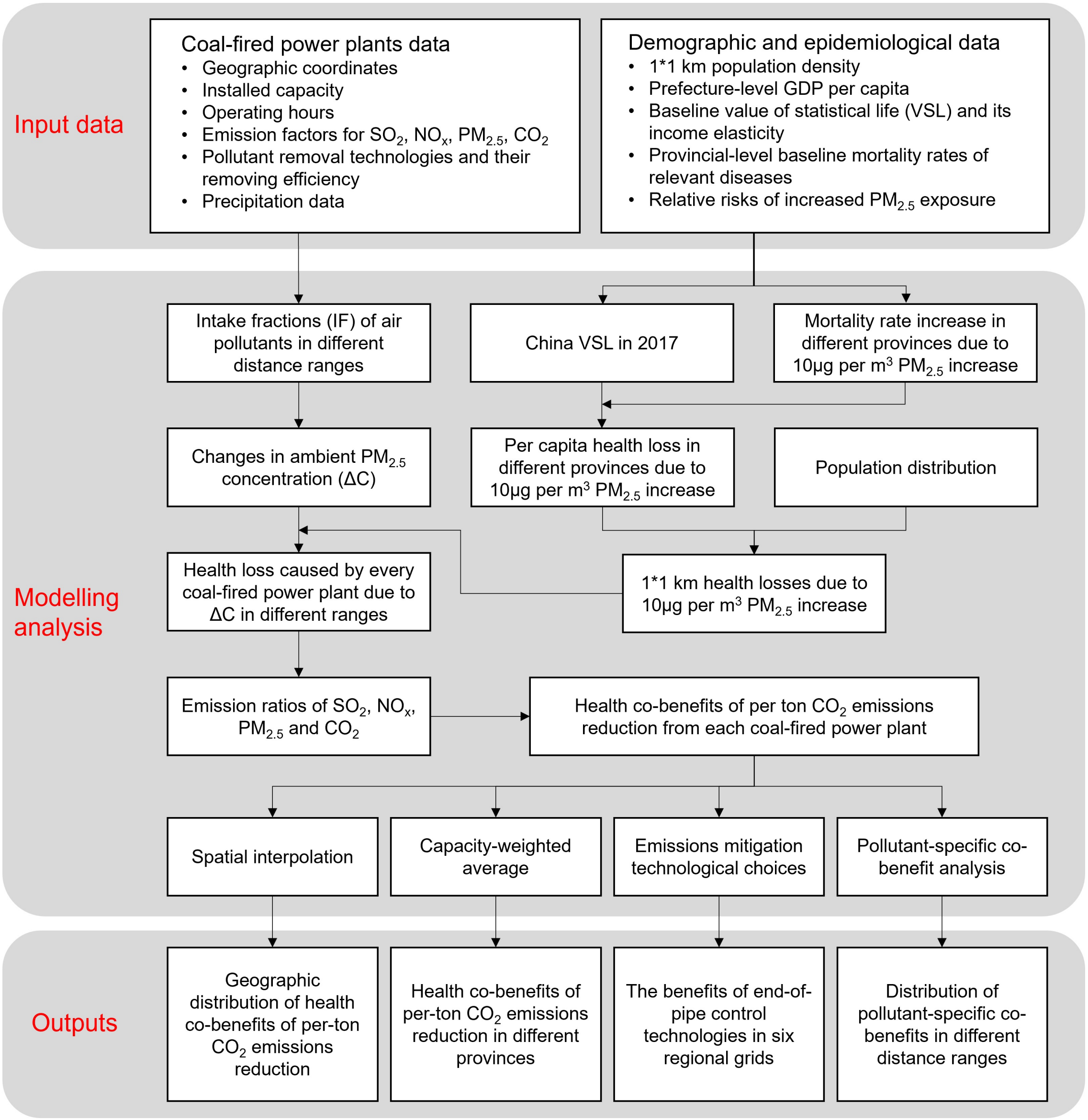

Supplementary Fig. 1 A flow chart of model inputs, calculation steps, and outputs.

Supplementary note 1:

The four major factors that influence the value of locational health co-benefits are: 1) the technological specifications of a power plant; 2) population within different ranges from a plant; 3) baseline mortality rates of diseases related to air pollution, including ischemic heart disease (IHD), chronic obstructive pulmonary disease (COPD), and lung cancer; and 4) local climatic conditions, most importantly precipitation levels.

For each specific plant, the relative importance of the four factors can vary significantly. To understand the relative importance of the four factors on average, we ran multiple

simulations to evaluate the national average effects of a 10% increase in each factor. (1) A 10% increase in population density within 500km from a power plant will increase the co-benefit value by 4.17% on average; since population size within 500km of a power plant ranges from 1.4 million to 443 million, population density change is a dominant factor that affects co-benefit value. (2) A 10% increase in annual precipitation will decrease the co-benefit value by roughly 3%, while annual precipitation in China ranges from 30mm to 2770mm. (3) A 10% increase in the emission factors of SO_2, NO_x, and $PM_{2.5}$ simultaneously will increase the co-benefit value by 10%, while a 10% increase in CO_2 emission factors will decrease the co-benefit value by 9.09%; changes in emission factors range between 100%-344%. (4) A 10% increase in baseline mortality rates of the three relevant diseases in the province where a power plant is located will increase the co-benefit value by 0.51%; changes in baseline mortality rates range between 100% to 232%. In summary, population density has the dominant impact on co-benefit values, and precipitation and technology specifications (represented by emission factors) have modest impacts on co-benefit values, while baseline mortality rate change has relatively small impact.

Supplementary note 2:

The results in Figure 1 are based on a universal value of statistical life (VSL) calculated from China's national per capita GDP in 2017. The locational co-benefit values will change with different assumptions. First, we re-estimate the co-benefits using VSLs based on GDP per capita at each of China's 337 prefecture-level cities (Supplementary Fig. 2). Compared to results in Figure 1, the co-benefit values in high income regions (such as Beijing, Tianjin, and Shanghai) increase slightly, while the values in in low income regions (such as Gansu, Guangxi, and Yunnan) decrease slightly. But overall, the patterns in the two figures are not significantly different from each other.

Second, a power plant can have many detrimental effects on adjacent areas other than $PM_{2.5}$ (e.g., coal ash and heavy metal deposition). Keeping other assumptions the same as Supplementary Fig. 2, we multiply the economic loss within 100km by three to take into account the other negative local impacts (see Methods and Supplementary Fig. 3). Compared to the results without local adjustments, while the general patterns are the same, the effects of the mega-cities are more prominent when local impacts are given higher weights. Particularly, the Beijing-Tianjin, Chengdu-Chongqing, and Hong Kong-Guangzhou-Shenzhen metropolitan areas show values significantly higher than their adjacent areas. From a regional planning perspective, policy makers can give higher weights to local impacts in order to avoid construction of coal plants in metropolitan areas.

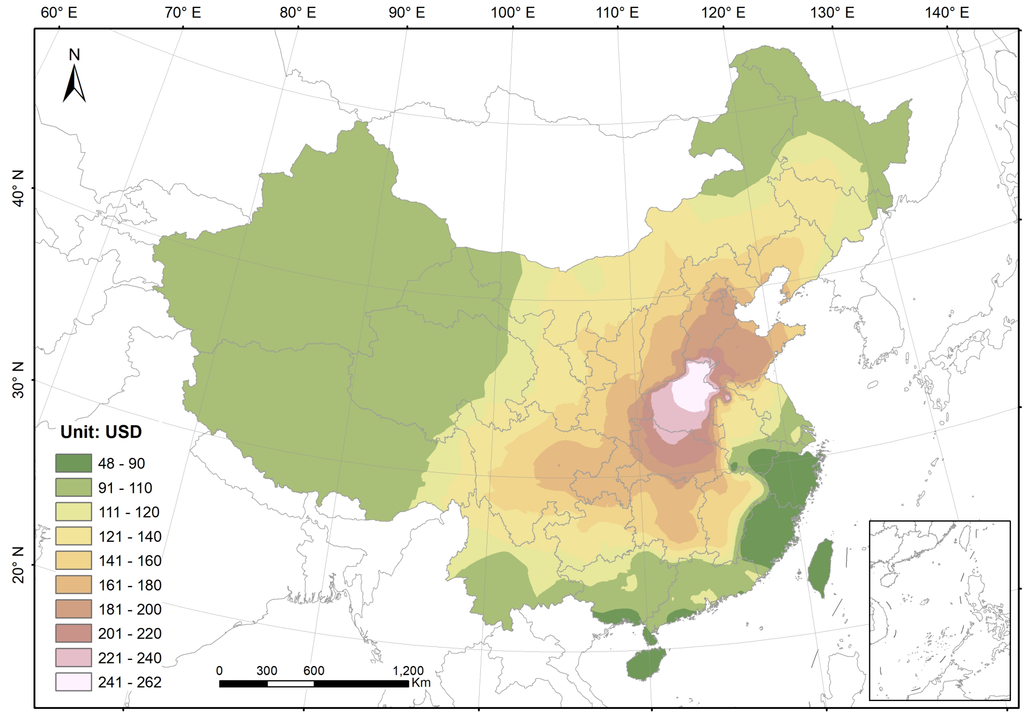

Supplementary Fig. 2 Health co-benefits of per-ton CO_2 emissions reduction based on GDP per capita at prefecture-level. (Unit is in USD).

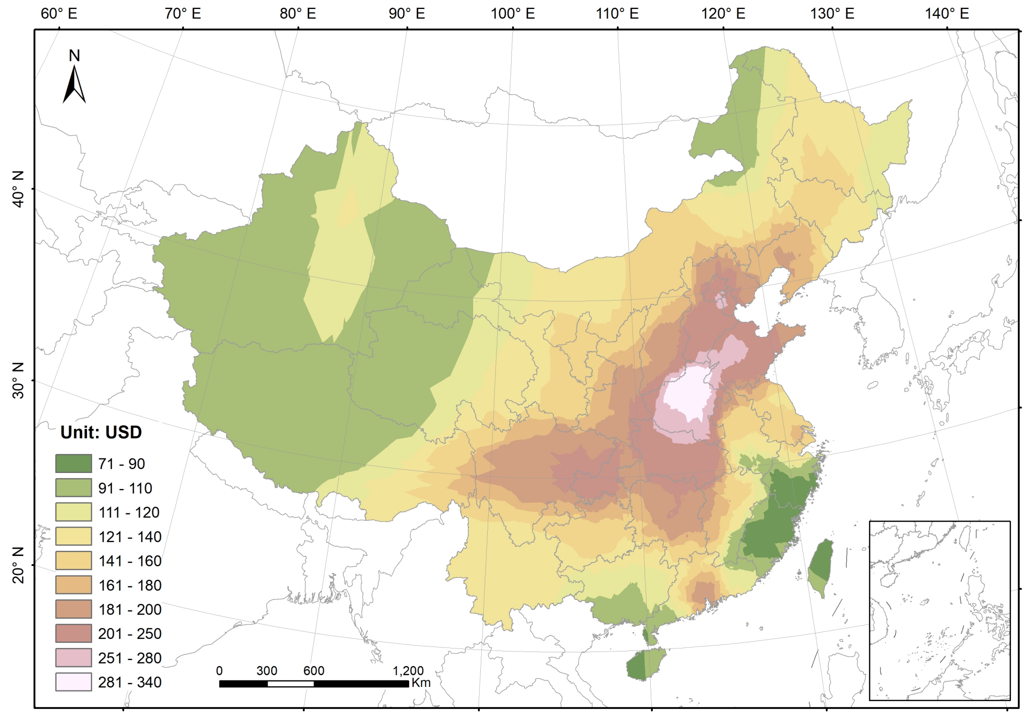

Supplementary Fig. 3 Health co-benefits of per-ton CO_2 emissions reduction when local impacts are given higher weights. (Unit is in USD).

Supplementary note 3:

The VSL used to calculate the results in the main text is based on: 1) China's national per capita GDP in 2017; 2) baseline VSL recommended by the United States Environmental Protection Agency (USEPA), which is 7.4 million USD per person in 2006 value; and 3) an income elasticity of 0.5.

We reviewed the VSL values used in similar studies, including: 1) Shindell et al. [1] employed the USEPA preferred VSL of $9.5 million for 2030, and used an elasticity of 0.4 to estimate country-specific VSLs based on each country's income per capita; 2) Li et al. [2] used the USEPA suggested VSL value and an elasticity of 0.4 to calculate VSLs for each Chinese province based on the ratios of GDP per capita, similar to the procedure in Shindell et al.; 3) West et al. [3] used both low VSLs and high VSLs: $1.8 million for Western Europe (OECD recommended value) as the benchmark low estimate, and $7.4 million for the U.S. (USEPA recommended value) as the benchmark high estimate, which are both adjusted to different world regions and into the future using an income elasticity of 0.5; 4) Cao et al. [4] estimated China's VSL through a contingent valuation study in six representative cities; they recommended a mean VSL value for China, which is 5.1 million CYN in 2019 price, or 725,000 USD in 2017 value.

Here we calculate co-benefits using alternative VSLs and elasticities: 1) China's national VSL based on the USEPA recommended benchmark VSL (7.4 million USD per person in 2006 value) and an income elasticity of 1 to reflect the impact of a relatively high elasticity; 2) China's national VSL based on Cao et al.'s contingent valuation study, which is 5.1 million CYN in 2019 price, or 725,000 USD in 2017 value; 3) prefecture-level VSLs based on local income, using the USEPA recommended benchmark VSL and an elasticity of 0.5 (corresponding to Supplementary Fig. 2); 4) same assumptions as (3) but giving higher weights to local impacts (corresponding to Supplementary Fig. 3). These results are presented in Supplementary Figures 4a-4d, respectively. The co-benefits spatial distribution maps corresponding to Supplementary Figures 4a and 4b are presented in Supplementary Figures 5 and 6, respectively. The results indicate that while different choices of VSLs and elasticities have significant impact on co-benefit values, they do not significantly change the ranking of the values. Therefore, choosing different VSLs and elasticities will not significantly change the priority order for coal plants phasing out or retrofitting, but it will change the ratios between carbon costs and air pollution costs and affect the stringency of coal restriction polices.

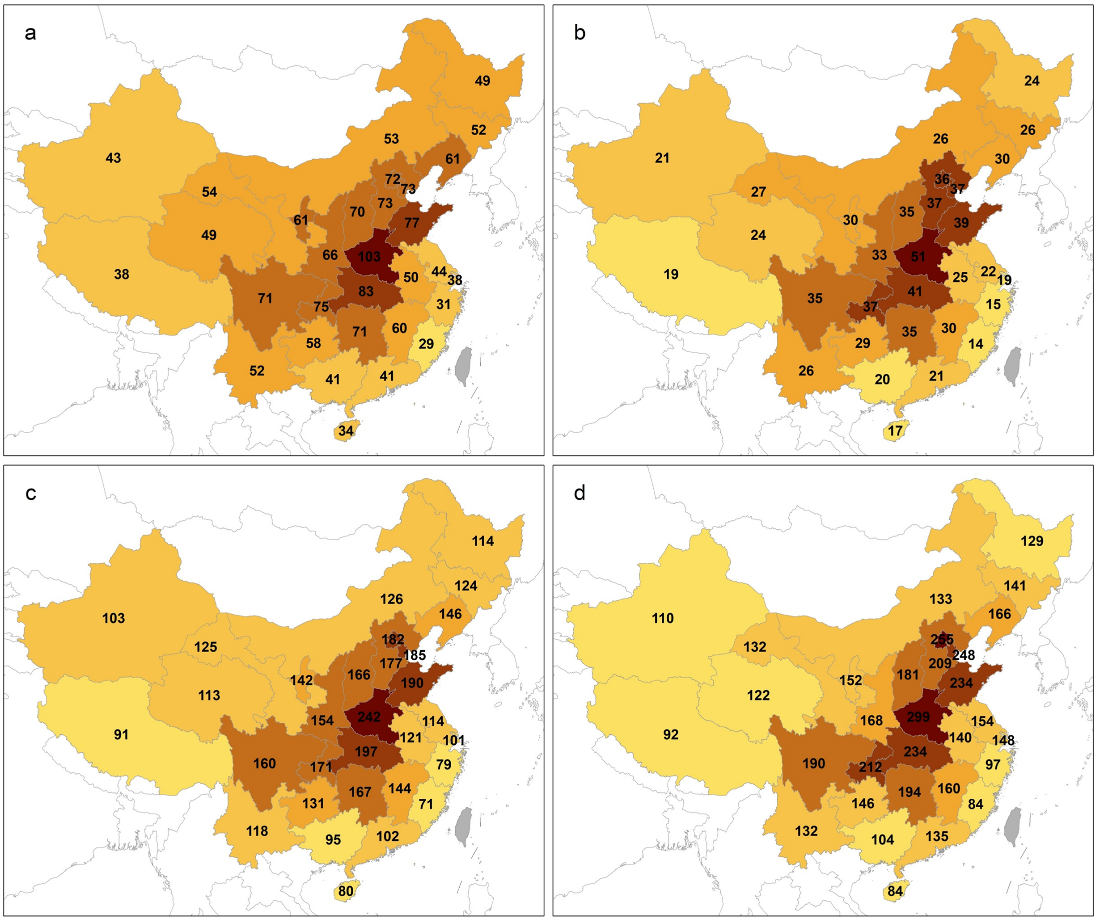

Supplementary Fig. 4 Provincial co-benefits based on alternative VSLs and elasticities. (Unit is in USD) a. national VSL based on the USEPA recommended benchmark VSL and an elasticity of 1; b. national VSL using 725,000 USD in 2017 value; c. prefecture-level VSLs based on local income, using the USEPA recommended benchmark VSL and an elasticity of 0.5; d. same assumptions as c but giving higher weights to local impacts.

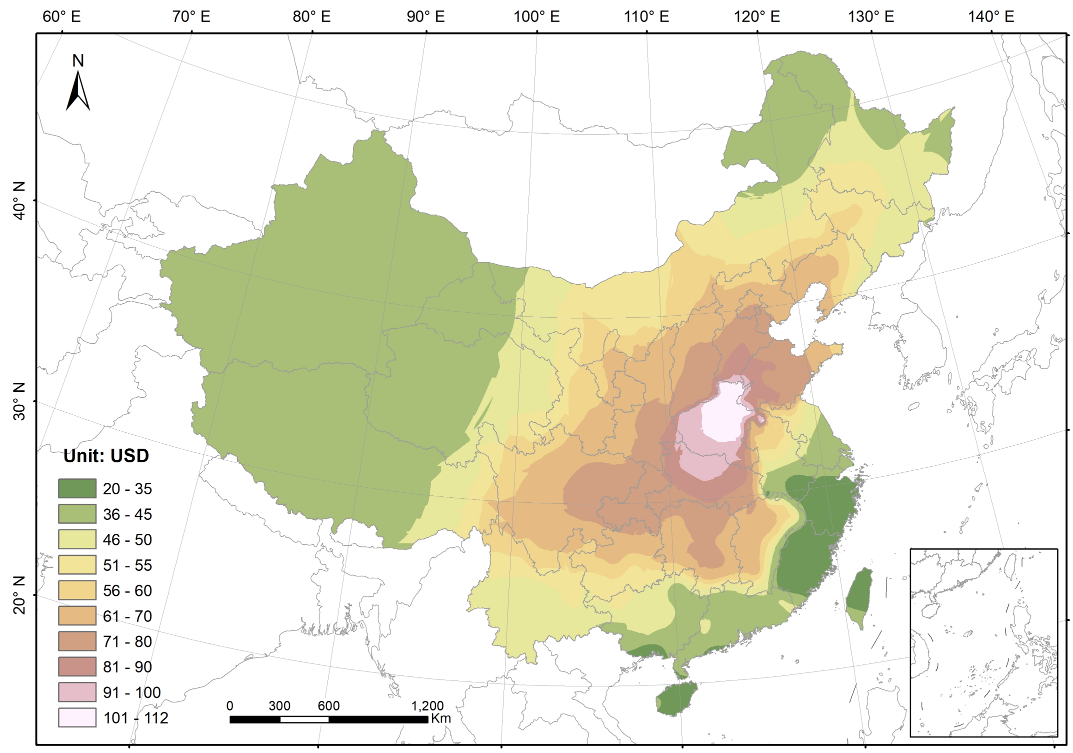

Supplementary Figure 5: Health co-benefits of per-ton CO_2 emissions reduction based on the USEPA recommended benchmark VSL and an elasticity of 1. (Unit is in USD).

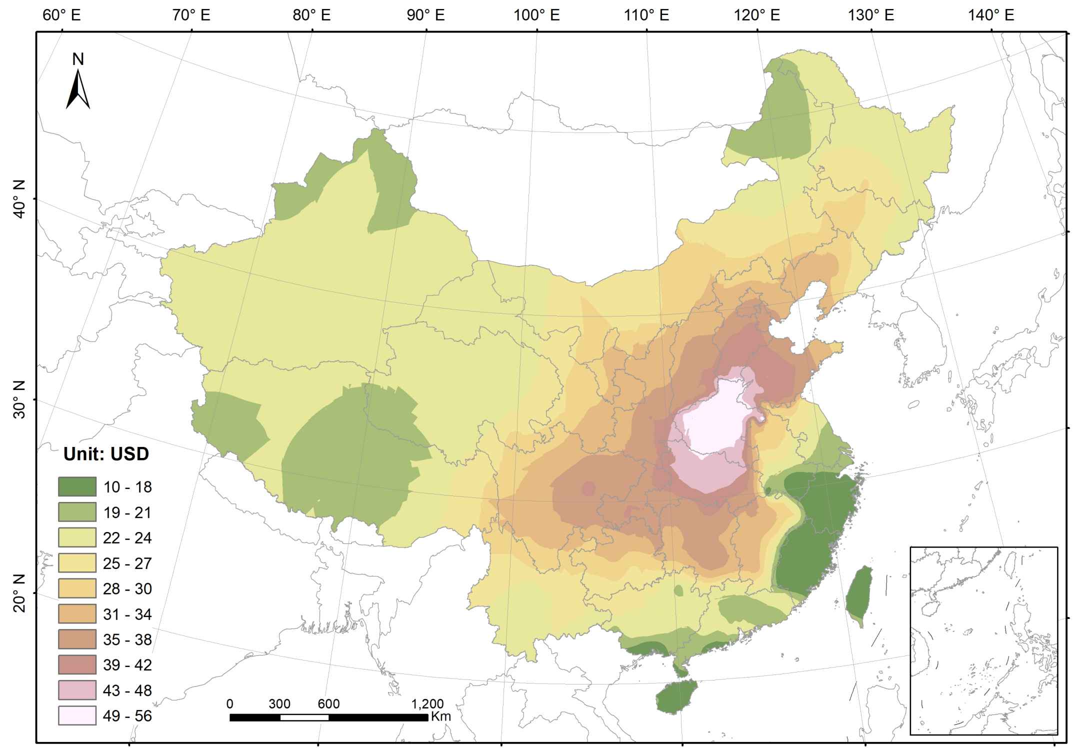

Supplementary Figure 6: Health co-benefits of per-ton CO_2 emissions reduction based on national VSL using 725,000 USD in 2017 value. (Unit is in USD).

Supplementary note 4:

Zhou et al. 2006 [5] study used data that are actual and representative, and their model coefficients were calibrated specifically for China's coal-fired power plants nationwide. They randomly selected one power plant located in each of 29 provincial administrative units in Mainland China. Thus these plants were distributed roughly evenly across the country and covered all the six regional grids. The model they used was CALPUFF, which is a multi-layer, multi-species non-steady-state air quality modelling system that simulates the effects of meteorological conditions on pollutant transport, transformation and removal (http://www.src.com/).

While the CALPUFF model takes into account seasonal changes of precipitation and winds, Zhou et al decided to choose population density and precipitation as independent variables to calculate IF coefficients, because they found that population density and precipitation have the strongest predictive power and can explain most of variations in exposure (R-squared >0.9), while average wind speed/directions and some other meteorological factors are either non-significant or relatively insubstantial to predict annual average impacts. Since population sizes within different ranges from a power plant are the independent variables in the regression, changes in population from the early 2000s to 2017 are taken into account by multiplying the 2017 population sizes and corresponding IF coefficients. In future studies, it is possible to estimate the IF coefficients with a larger sample to improve accuracy.

We created a national inventory for pollutants emissions from all coal-fired power plants in China, and use GEOS-Chem and intake fractions (IF) models to simulate the overall impacts of these power plants in 2017, in terms of the increase in exposure to $PM_{2.5}$ and the corresponding values of health losses. Supplementary Figures 7 and 8 present the distribution of health losses in 1*1km grids, based on IF and GEOS-Chem models, respectively. The simulation results show that national total health losses due to coal-fired plants in 2017 was 460.1 billion USD based on IF model, and was 426.4 billion USD based on GEOS-Chem; while the latter was 7.3% lower than the former, the maximum value from GEOS-Chem model results was higher than that of the IF model.

We also compared GEOS-Chem simulation results with the actual observation data from 1444 air quality monitoring stations across China (Supplementary Fig. 9). The result indicates that GEOS-Chem tends to underestimate $PM_{2.5}$ concentrations in remote provinces (the blue dots), while overestimate $PM_{2.5}$ concentrations in central provinces (red dots). More discussion of the model comparisons is presented in the Results Section in the main text.

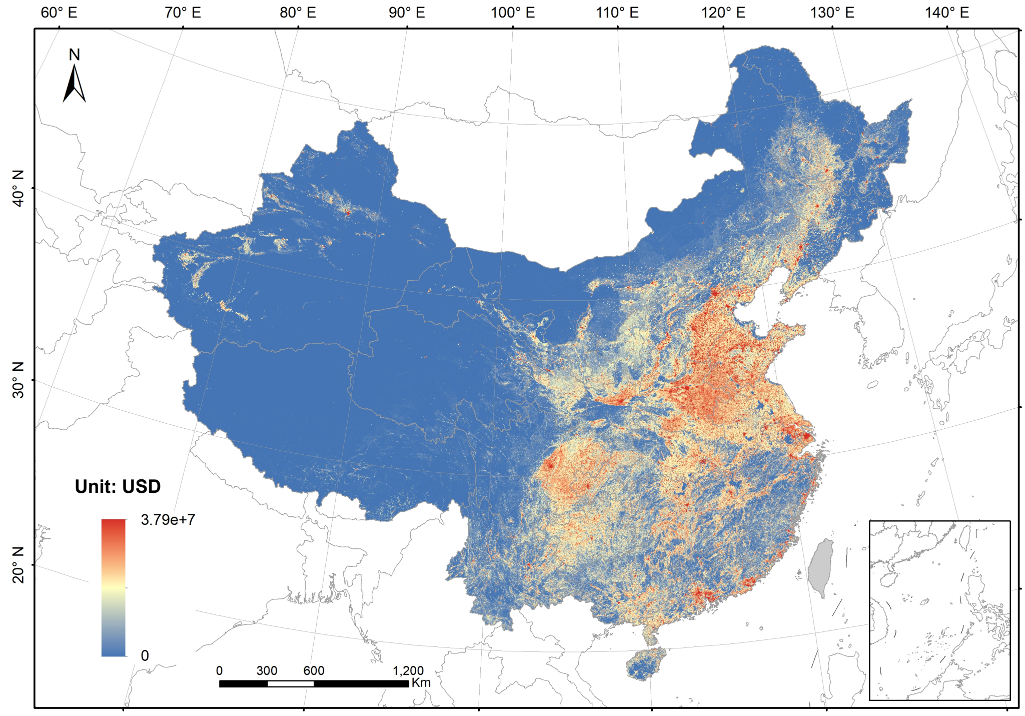

Supplementary Fig. 7 Distribution of health losses due to air pollution caused by coal-fired power plants in 2017, based on intake fractions (IF) model. For each 1x1 km grid, the value represents the total health loss suffered by the population living in the grid, induced by air pollutants emitted from all coal-fired power plants in China in 2017. (Unit is in USD)

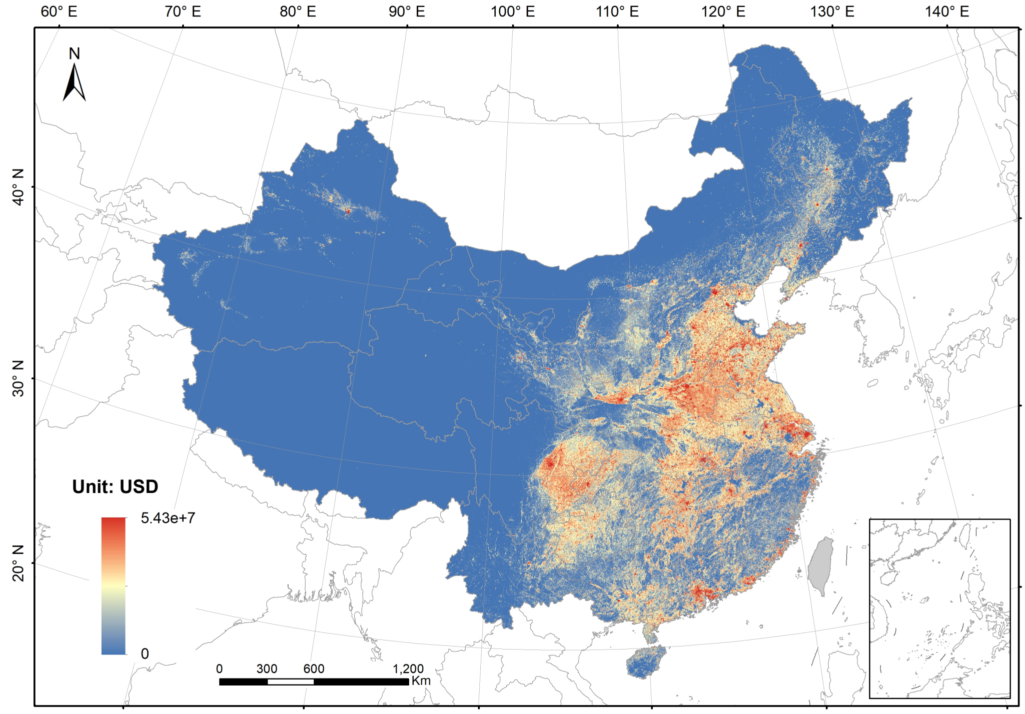

Supplementary Fig. 8 Distribution of economic losses due to air pollution caused by coal-fired power plants in 2017, based on GEOS-Chem model. For each 1x1 km grid, the value represents the total health loss suffered by the population living in the grid, induced by air pollutants emitted from all coal-fired power plants in China in 2017. (Unit is in USD)

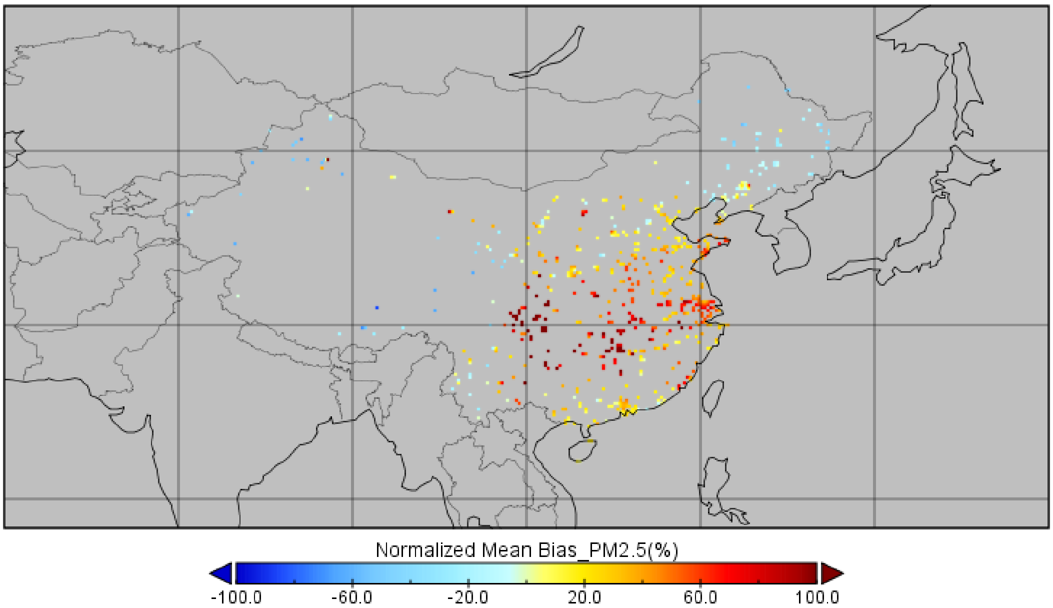

Supplementary Fig. 9 Comparison of GEOS-Chem simulated annual $PM_{2.5}$ concentration values with the actual observation data. Normalized mean bias is calculated as (simulated value-observed value)/observed value.

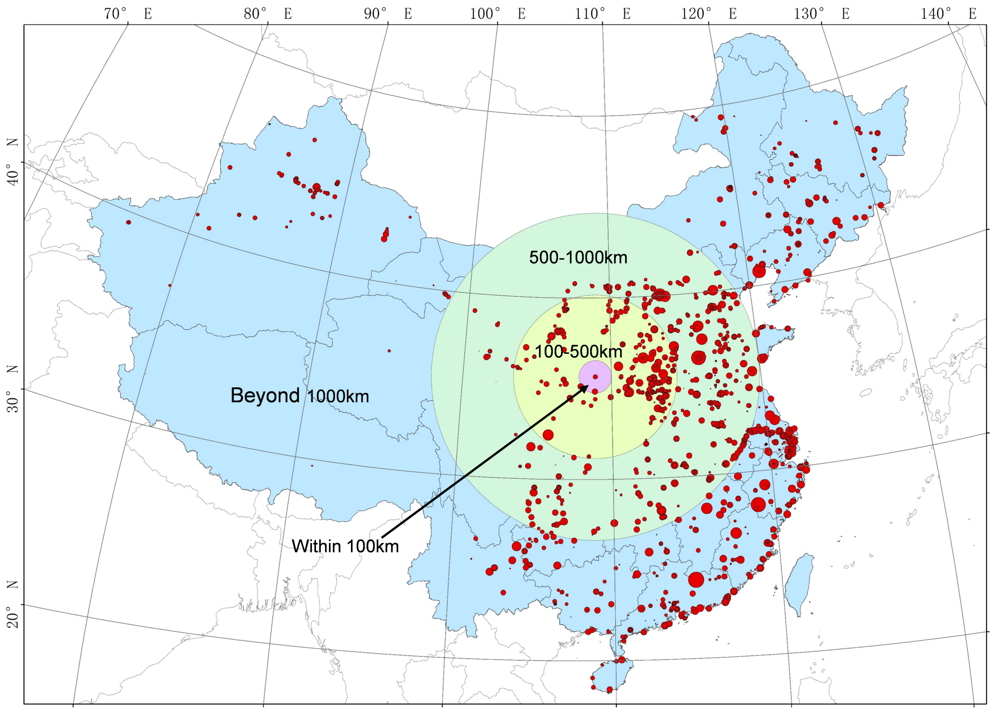

Supplementary Fig. 10 Distribution of China's coal-fired power plants. Each red dot represents a coal-fired power plant, and the size of the dot is proportional to the plant's installed capacity. For each power plant, the affected regions are divided into four ranges according to the distance from the power plant: within 100km, 100-500km, 500-1000km, and beyond 1000km.

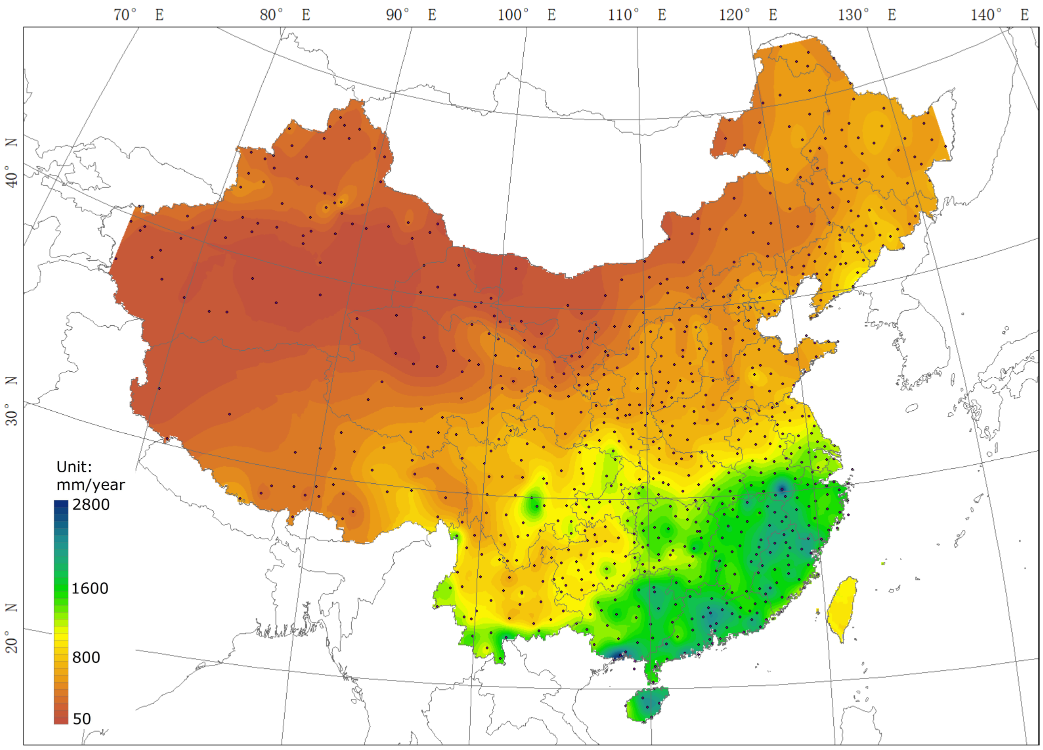

Supplementary Fig. 11 Average annual precipitation between 2011-2016. The data is calculated based on hourly meteorological monitoring data from 839 stations (round dots) across China.

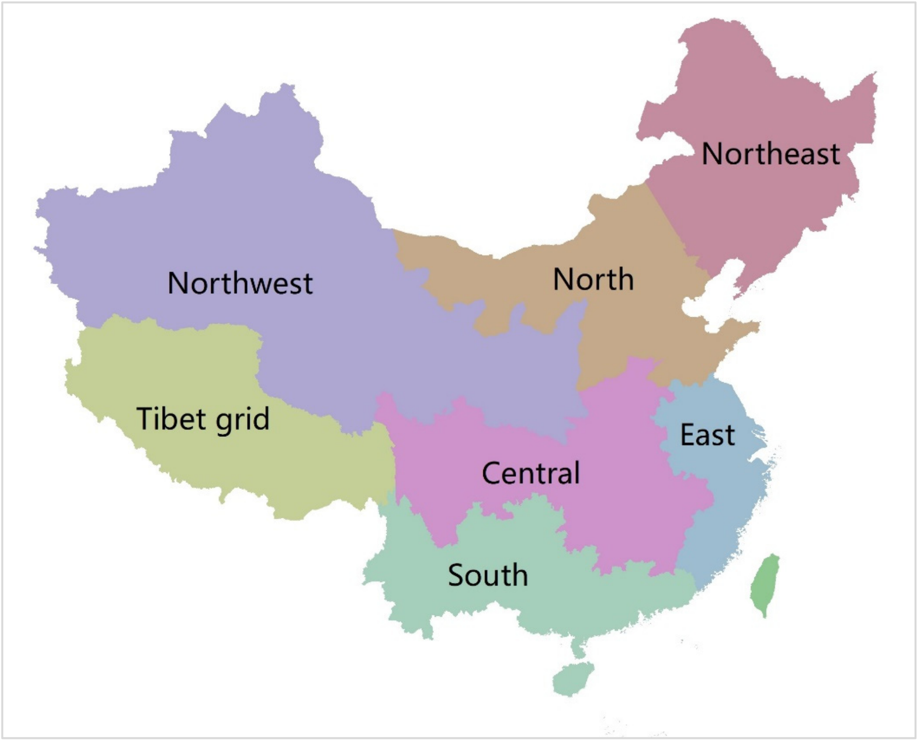

Supplementary Fig. 12 Six major regional electricity grids in China. Tibet grid is connected with Northwest grid.

Supplementary Table 1. Age-standardised rates of death (per 100,000 people) for ischemic heart disease (IHD), chronic obstructive pulmonary disease (COPD), and lung cancer in China and its provinces for men and women in 2013.

Province	Men Ischaemic heart disease	Men COPD	Men Lung cancer	Women Ischaemic heart disease	Women COPD	Women Lung cancer	Sex ratio
**Anhui**	118.3	91.2	62.3	95.9	57.9	20.7	104.9
**Beijing**	149.5	37.7	51.5	104.3	23.7	23.4	109.45
**China**	**137.6**	**99.6**	**60.1**	**95.1**	**61.9**	**21.9**	**105.02**
**Chongqing**	97.6	171.7	75.3	72.5	103.7	24.9	100.6
**Fujian**	101	119.9	63.5	65.1	58	18	105.71
**Gansu**	121.8	175.3	24.3	80	141.1	10.7	106.18
**Guangdong**	137.2	95.7	55.8	84.4	52.5	18.5	113.51
**Guangxi**	158.1	134	63.6	90.9	86.3	19.6	105.57
**Guizhou**	100.5	196	54.7	73.6	131.8	18.1	107.12
**Hainan**	104.8	101.6	46	59.6	57.3	11.8	110.47
**Hebei**	186	49.1	60.7	120.1	28.6	20.3	102.15
**Heilongjiang**	218.9	50	71.2	165.8	40.8	38.9	101.59
**Henan**	172.9	89.5	53.2	126	50.3	20.2	103.99
**Hubei**	120.8	101.6	60	90.4	57.4	19.1	104.12
**Hunan**	143.2	97.9	54.1	95.7	58.3	16.8	102.98
**Inner Mongolia**	198	80.1	53.1	139.8	59.3	24.7	104.32
**Jiangsu**	89.5	80.9	54.2	62.9	48.7	19.1	103.01
**Jiangxi**	125.2	104.1	62	88	62.1	20.6	106.12
**Jilin**	214.8	39.2	57.2	156.6	29	28.4	102.02
**Liaoning**	194.5	43.4	79.1	126.2	31.9	35.2	100.45
**Ningxia**	181.1	80.6	40.8	128.2	68.3	18.6	106.16
**Qinghai**	162.1	162.8	31.7	132.1	131.7	17.1	109.29
**Shaanxi**	159	68.2	41.1	116.9	42.2	15.4	102.59
**Shandong**	175.8	79.8	73.1	115.8	49.6	26	104.45
**Shanghai**	60.5	68.2	52.2	44.8	31.7	17.9	108.37
**Shanxi**	146.3	72.1	57.7	104.8	37.8	19.9	107.49
**Sichuan**	89	173.4	69.8	63.3	120.5	26	100.89
**Tianjin**	153.7	34	53.8	119.6	26.6	29.6	120.43
**Tibet**	107.8	63.6	7.4	87.8	44.9	4.6	102.33
**Xinjiang**	209.6	119.7	40.3	157.6	99.1	19.4	104.12
**Yunnan**	118.5	175.6	48.3	76.3	104.2	16.5	104.98
**Zhejiang**	50.2	86.9	60.7	38.7	55.5	18.2	107.37

Notes:

1. The disease-specific mortality data is based on Zhou and colleagues' Global Burden of Disease (GBD) project study [6] .

2. The sex ratio data is based on China's National Statistics Yearbook.

Supplementary Table 2. Coefficients for intake fractions with population, distances, and precipitation as independent variables.

Intake fraction (dependent variable)	Independent variables (coefficients and standard errors)				
	Population within 100 km	Population between 100 and 500 km	Population between 500 and 1000 km	Population beyond 1000 km	Precipitation
SO_4	2.4E-8 (3.7E-8)	7.9E-9 (4.5E-9)	6.9E-9 (2.2E-9)	2.6E-9 (7.1E-10)	-1.2E-9 (4.0E-10)
NO_3	4.3E-8 (3.7E-8)	1.3E-8 (4.4E-09)	3.5E-9 (2.2E-9)	2.5E-9 (7.1E-10)	-1.9E-9 (4.0E-10)
PM_1	1.5E-7 (7.8E-8)	2.3E-8 (9.3E-9)	1.1E-8 (4.6E-9)	3.9E-9 (1.5E-9)	-1.7E-9 (8.4E-10)
PM_3	1.4E-7 (6.7E-8)	1.7E-8 (8.1E-9)	6.4E-9 (3.9E-9)	3.0E-9 (1.3E-9)	-2.4E-9 (7.2E-10)

Notes:

1. Data in this table is based on Zhou et al's[5] calculation of intake fractions for primary pollutants (primary particles), secondary sulfate (SO_4) (defined as ammonium sulfate inhaled per unit of sulfur dioxide emissions) and secondary nitrate (NO_3) (defined as ammonium nitrate inhaled per unit of nitrogen oxides emissions).
2. For modelling purpose, in Zhou et al's study, PM_1 was defined to be particles of precisely 1 μm in aerodynamic diameter, with parallel definitions for PM_3. This definition is different from common definitions of $PM_{2.5}$. According to Lu and Yao's study on characteristics of particulate matters from coal plants in China[7], we use the mean of intake fractions of PM_1 and PM_3 in Zhou et al's study to approximate intake fractions of primary PM with aerodynamic diameters less than 2.5 μm.
3. Population variable in millions of people.
4. Precipitation variable in mm per year.

Supplementary Table 3. Regional grid level average emission factors for CO_2, SO_2, NO_x, and $PM_{2.5}$ per kWh of electricity generation in 2010.

**Grid**	**Sulfur content (%)**	**Emission factors (g/kWh)**			
		**SO_2**	**NO_x**	**$PM_{2.5}$**	**CO_2**
North	1.00	2.45	2.79	0.26	914.7
Northeast	0.51	2.23	3.32	0.55	1042.9
East	0.69	1.26	2.28	0.16	877.3
Central	1.18	3.27	2.73	0.34	821.7
Northwest	0.98	3.44	2.78	0.27	956.7
South	1.32	3.41	2.56	0.20	904.4

Note:
The data is according to Liu et al. study based on the Multi-resolution Emission Inventory for China (MEIC) [8].

Supplementary Table 4. Changes in national emission factors from 2010 to 2017.

	Total generation (10^8 kwh)	Total SO_2 emission (Mn ton)	Total NO_x Emission (Mn ton)	Total $PM_{2.5}$ emission (Mn ton)	Coal consumption (g/kWh)	SO_2 emission (g/kWh)	NO_x emission (g/kWh)	$PM_{2.5}$ emission (g/kWh)
2010	34166	7.8	8.6	0.8	333	2.28	2.52	0.23
2017	45558	1.8	4.2	0.6	309	0.40	0.92	0.13
2017/2010 ratio					92.8%	17.3%	36.6%	56.2%

Notes:
1. Total emissions in 2010 and 2017 are according to Zheng et al. study based on MEIC[9].
2. Total generation and coal consumption per kWh data are based on China Electricity Council's annual reports[10,11].

Supplementary References

1 Shindell, D. *et al.* Simultaneously mitigating near-term climate change and improving human health and food security. *Science* **335**, 183-189 (2012).

2 Li, M. *et al.* Air quality co-benefits of carbon pricing in China. *Nature Climate Change* **8**, 398-403, doi:10.1038/s41558-018-0139-4 (2018).

3 West, J. J. *et al.* Co-benefits of Global Greenhouse Gas Mitigation for Future Air Quality and Human Health. *Nature Climate Change* **3**, 885-889, doi:10.1038/NCLIMATE2009 (2013).

4 Cao, C. *et al.* Estimating the Value of Statistical Life in China: A Contingent Valuation Study in Six Representative Cities. Preprint at https://assets.researchsquare.com/files/rs-199197/v1_stamped.pdf?c=1612551473. (2021).

5 Zhou, Y., Levy, J. I., Evans, J. S. & Hammitt, J. K. The influence of geographic location on population exposure to emissions from power plants throughout China. *Environ Int* **32**, 365-373, doi:10.1016/j.envint.2005.08.028 (2006).

6 Zhou, M. *et al.* Cause-specific mortality for 240 causes in China during 1990–2013: a systematic subnational analysis for the Global Burden of Disease Study 2013. *The Lancet* **387**, 251-272 (2016).

7 Lu, S. & Yao, D. Study on emissions characteristics of particles in coal-burning power plant (in Chinese). *Environmental Pollution & Control* **8** (2010).

8 Liu, F. *et al.* High-resolution inventory of technologies, activities, and emissions of coal-fired power plants in China from 1990 to 2010. *Atmospheric Chemistry and Physics* **15**, 13299-13317 (2015).

9 Zheng, B. *et al.* Trends in China's anthropogenic emissions since 2010 as the consequence of clean air actions. *Atmospheric Chemistry and Physics* **18**, 14095-14111 (2018).

10 China Electricity Council. *China's electric power industry annual development report (in Chinese, Zhongguo dianli hangye niandu fazhan baogao)*. (China Market Press, 2011).

11 China Electricity Council. *China's electric power industry annual development report (in Chinese, Zhongguo dianli hangye niandu fazhan baogao)*. (China Market Press, 2018).
