# Supplementary material for: Location-specific co-benefits of carbon emissions reduction from coal-fired power plants in China
Source: Nat Commun. 2021 Nov 29;12:6948. doi: 10.1038/s41467-021-27252-1 (PMC8629986; doi:10.1038/s41467-021-27252-1)
Supplement: Supplementary file 3 — Reporting Summary [file 41467_2021_27252_MOESM3_ESM.pdf]

nature research

Corresponding author(s): Pu Wang, Yi Wang

Last updated by author(s): Nov 3, 2021

Reporting Summary

Nature Research wishes to improve the reproducibility of the work that we publish. This form provides structure for consistency and transparency in reporting. For further information on Nature Research policies, see our Editorial Policies and the Editorial Policy Checklist.

Statistics

For all statistical analyses, confirm that the following items are present in the figure legend, table legend, main text, or Methods section.

n/a	Confirmed	
☐	☒	The exact sample size (n) for each experimental group/condition, given as a discrete number and unit of measurement
☒	☐	A statement on whether measurements were taken from distinct samples or whether the same sample was measured repeatedly
☒	☐	The statistical test(s) used AND whether they are one- or two-sided<br>*Only common tests should be described solely by name; describe more complex techniques in the Methods section.*
☒	☐	A description of all covariates tested
☒	☐	A description of any assumptions or corrections, such as tests of normality and adjustment for multiple comparisons
☐	☒	A full description of the statistical parameters including central tendency (e.g. means) or other basic estimates (e.g. regression coefficient) AND variation (e.g. standard deviation) or associated estimates of uncertainty (e.g. confidence intervals)
☒	☐	For null hypothesis testing, the test statistic (e.g. F, t, r) with confidence intervals, effect sizes, degrees of freedom and P value noted<br>*Give P values as exact values whenever suitable.*
☒	☐	For Bayesian analysis, information on the choice of priors and Markov chain Monte Carlo settings
☒	☐	For hierarchical and complex designs, identification of the appropriate level for tests and full reporting of outcomes
☒	☐	Estimates of effect sizes (e.g. Cohen's d, Pearson's r), indicating how they were calculated

Our web collection on statistics for biologists contains articles on many of the points above.

Software and code

Policy information about availability of computer code

Data collection	No software was used in data collection.
Data analysis	ArcGIS 10.7 is used in modelling of location-specific pollution exposure and intake fraction. GEOS-Chem model (GCHP 13.0.2 version) is used to statistically test the accuracy of the Intake Fractions model.

For manuscripts utilizing custom algorithms or software that are central to the research but not yet described in published literature, software must be made available to editors and reviewers. We strongly encourage code deposition in a community repository (e.g. GitHub). See the Nature Research guidelines for submitting code & software for further information.

Data

Policy information about availability of data

All manuscripts must include a data availability statement. This statement should provide the following information, where applicable:

- Accession codes, unique identifiers, or web links for publicly available datasets
- A list of figures that have associated raw data
- A description of any restrictions on data availability

Source data are provided with this paper. Other data that support the findings of this study are available from the corresponding author upon reasonable request.

Field-specific reporting

Ecological, evolutionary & environmental sciences study design

All studies must disclose on these points even when the disclosure is negative.

Study description	We establish an interdisciplinary approach to assess facility level climate-air quality co-benefits at specific locations. Our model takes advantage of a complete coal-fired power plants dataset, the latest fine-scale demographic and health data, and intake fractions of air pollutants calibrated specifically for coal plants in China. We calculated the locational health co-benefits of per ton CO2 emissions reduction based on the decrease in air pollution related mortality. Our analyses capture technological features, fuel quality, and the specific location of each individual power plant, as well as the relevant demographic, economic, weather, and epidemiological information of affected regions.
Research sample	Our coal-fired power plants dataset included 2475 power plants (for some plants each generation unit is a single entry), with total installed capacity of 914GW, which represents 93% of China's coal fleet in 2017.
Sampling strategy	We aimed to collect nationwide power plant information as complete as possible. Our final dataset covers 93% of China's coal fleet in 2017.
Data collection	The information on geographic coordinates, technology type, and installed capacity of each individual coal-fired power plant in China was extracted from the open-source dataset, "Existing coal plants in China" compiled by Global Energy Monitor's Global Coal Plant Tracker. Population distribution data are based on the 1x1 km global population grid data developed by Oak Ridge National Laboratory's LandScan project. China's prefecture-level GDP data were extracted from Chinese statistical yearbooks for the year 2017. Province-level mortality rates for diseases associated with ambient air pollution were based on Zhou et al.'s 2016 study, which was part of the Global Burden of Disease (GBD) project. Precipitation data between 2011-2016 are based on hourly meteorological monitoring data from 839 stations across China.
Timing and spatial scale	We collected China's national power plants data up to 2017. Prefecture-level annual socioeconomic data was in 2017. Hourly precipitation data was between 2011-2016.
Data exclusions	No data were excluded from the analyses.
Reproducibility	We ran the models multiple times and the results have been reproductible.
Randomization	This is not relevant to our study, since our analyses were applied to the complete dataset.
Blinding	This is not relevant to our study, since our analyses were applied to the complete dataset.

Did the study involve field work? ☐ Yes ☒ No

Reporting for specific materials, systems and methods

We require information from authors about some types of materials, experimental systems and methods used in many studies. Here, indicate whether each material, system or method listed is relevant to your study. If you are not sure if a list item applies to your research, read the appropriate section before selecting a response.

Materials & experimental systems

n/a	Involved in the study	
☒	☐	Antibodies
☒	☐	Eukaryotic cell lines
☒	☐	Palaeontology and archaeology
☒	☐	Animals and other organisms
☒	☐	Human research participants
☒	☐	Clinical data
☒	☐	Dual use research of concern

Methods

n/a	Involved in the study	
☒	☐	ChIP-seq
☒	☐	Flow cytometry
☒	☐	MRI-based neuroimaging
